# Genotype-Specific Distribution of High-Risk Human Papillomavirus (HPV) and Microbial Co-Detections in HPV-Positive Women from Southern Croatia

**DOI:** 10.3390/biomedicines13092100

**Published:** 2025-08-28

**Authors:** Vanja Kaliterna, Tomislav Meštrović, Mirjana Čorić-Mesarić, Ivana Božičević

**Affiliations:** 1 Teaching Institute for Public Health of Split and Dalmatia County, 21000 Split, Croatia; 2 Faculty of Health Sciences, University of Split, 21000 Split, Croatia; 3 University Centre Varaždin, University North, 42000 Varaždin, Croatia; 4 Institute for Health Metrics and Evaluation, University of Washington, Seattle, WA 98105, USA; 5 WHO Collaborating Centre for HIV Strategic Information, Andrija Štampar School of Public Health, School of Medicine, 10000 Zagreb, Croatia; 6 School of Medicine, University of Zagreb, 10000 Zagreb, Croatia

**Keywords:** human papillomavirus (HPV), high-risk HPV genotypes, cervico-vaginal microbiota, sexually transmitted infections, HPV genotyping, epidemiology, Croatia

## Abstract

**Background/Objectives**: High-risk human papillomavirus (HPV) is the principal etiological agent of cervical cancer, with distinct genotype-specific oncogenic potential. While HPV type 16 is most frequently implicated in carcinogenesis, the role of other genotypes and their interaction with sexually transmitted infections and cervico-vaginal dysbiosis is gaining recognition. This study aimed to assess the genotype-specific distribution of high-risk HPV among HPV-positive women from Southern Croatia and examine associations with age and co-infections with selected microbial pathogens. **Methods**: We conducted a retrospective cross-sectional study on 1211 HPV-positive women (out of 3098 tested) from Split and Dalmatia County between 2023 and 2024. Cervico-vaginal swabs were tested using molecular and culture-based methods for 14 high-risk HPV genotypes and several pathogens, including *Chlamydia trachomatis*, *Mycoplasma genitalium*, *Mycoplasma hominis*, *Ureaplasma urealyticum*, *Gardnerella vaginalis*, and other microorganisms. In the analysis, each detected HPV genotype was also treated as a distinct line-level observation. Genotypes were grouped by phylogenetic and carcinogenic profiles, and statistical analyses—including chi-square tests and multinomial logistic regression—were performed to evaluate associations with age and co-infections. **Results**: Among high-risk HPV-positive women, the most frequently detected high-risk HPV genotypes were HPV 16 (23.3%), HPV 31 (22.4%), and HPV 51 (13.5%). Notably, HPV 18 was less prevalent (8.1%) and occurred at a similar frequency to HPV 58 and 68. Although younger age was significantly associated with high-risk HPV positivity (*p* < 0.001), no significant differences in HPV genotype group distribution were observed between age groups; however, *C. trachomatis* and *Streptococcus agalactiae* were significantly more prevalent in women aged ≤29 years (*p* < 0.001 and *p* = 0.029, respectively). Multinomial regression revealed that *C. trachomatis* was negatively associated with 16-related and lower-risk genotypes, while *G. vaginalis* showed a positive association with 16-related types. **Conclusions**: There is a complex interplay between high-risk HPV genotypes and microbial co-infections, which means the broader cervico-vaginal microbiome has to be considered in HPV risk assessment. The findings highlight the need for extended genotyping and microbial screening to inform regional prevention strategies.

## 1. Introduction

Human papillomavirus (HPV) is a widespread sexually transmitted infection and the principal cause of cervical cancer, detected in 99.7% of all cervical malignancies, and responsible for 10 million disability-adjusted life years (DALYs) per year on a global level [1,2,3]. HPV types 16 and 18 are implicated in approximately 70% of cervical cancer cases worldwide, yet over a dozen other high-risk types are also known to contribute to disease development [4,5,6]. While most HPV infections are transient and cleared by the immune system, persistent infection with high-risk types can lead to high-grade lesions and, ultimately, invasive cancer [7,8]. The distribution of HPV genotypes shows considerable geographic variation [9,10,11], highlighting the need for region-specific data to guide screening strategies and vaccination policies.

Recent studies have emphasized the clinical value of extended HPV genotyping, which provides detailed information on individual high-risk HPV types beyond HPV 16 and 18 [12,13]. This approach supports refined risk stratification, as some genotypes within the commonly used “12 other” pooled category (such as HPV 31, 33, 35, 52 and 58) carry a higher risk for progression to cervical intraepithelial neoplasia grade 3 or worse (≥CIN 3) [14,15]. Consequently, extended genotyping is increasingly advocated in international guidelines to improve patient management and ensure that women at greatest risk receive timely and appropriate care [16]. This is especially relevant for countries like the Republic of Croatia, where data on individual genotype distribution and their clinical implications remain limited.

In addition to virological factors, the composition of the cervico-vaginal microbiota and the presence of co-infections with other sexually transmitted pathogens have been recognized as important modulators of HPV infection dynamics. Pathogens and colonizers, such as *Chlamydia trachomatis* (*C. trachomatis*), *Neisseria gonorrhoeae* (*N. gonorrhoeae*), *Ureaplasma urealyticum* (*U. urealyticum*), *Gardnerella vaginalis* (*G. vaginalis*) and *Trichomonas vaginalis* (*T. vaginalis*), may impair epithelial integrity or alter local immune responses—potentially promoting viral persistence and disease progression [17,18,19]. Understanding these microbial interactions is, therefore, essential for identifying women at increased risk and for developing comprehensive screening and prevention strategies that account for the broader microbial context of HPV infection [19].

Previous research conducted in Southern Croatia suggested that the presence of specific bacteria—such as those from the genera *Ureaplasma*, *Chlamydia* and *Gardnerella*—may increase the likelihood of HPV infection and accelerate progression to cervical dysplasia [20]. However, such studies often relied on pooled HPV detection or limited genotyping; as a result, they are unable to distinguish the risk profiles of individual HPV types or fully explore how specific microbial patterns may influence different genotype groups.

This study aimed to address this gap by analyzing the distribution of high-risk human HPV genotypes in HPV-positive women from Split and Dalmatia County over a one-year period, and evaluating their associations with age and co-infections with selected sexually transmitted pathogens and indicators of cervico-vaginal dysbiosis. By categorizing HPV genotypes based on their phylogenetic and carcinogenic characteristics, and linking them to microbiological findings and key demographic variables such as age, we aim to contribute to a more nuanced understanding of HPV epidemiology and its microbial context.

## 2. Materials and Methods

A retrospective study approach was utilized to analyze the distribution of HPV genotypes in HPV-positive samples in a one-year period between 2023 and 2024. The study encompassed a total of 1211 HPV-positive women residing in Split and Dalmatia County, whose samples were collected over the course of one year in an outpatient setting at the Teaching Institute for Public Health of Split and Dalmatia County. Each woman underwent a gynecological examination, during which two cervico-vaginal swabs were collected—one for culture purposes and the other for molecular diagnostic testing targeting genital mycoplasmas, *C. trachomatis*, *T. vaginalis*, *N. gonorrhoeae* and high-risk HPV. Samples were collected using Amies medium (Copan Diagnostics, Brescia, Italy) for bacteriological analysis and eNAT medium (Copan Diagnostics, Brescia, Italy) for molecular diagnostics to ensure proper preservation and transport. The age of participants was noted to support epidemiological analysis and subgroup comparisons. All participants were informed about the procedures and provided verbal consent that was recorded in the patient medical record, agreeing that their results could be used anonymously for the purposes of this study.

Bacterial cultivation was performed using standard microbiological techniques. Vaginal and cervical swabs were inoculated on a range of selective and non-selective culture media suitable for detecting common aerobic and facultative anaerobic bacteria, yeasts, and microorganisms associated with vaginal dysbiosis. Incubation was carried out under appropriate atmospheric conditions, and microbial identification was performed based on colony morphology, biochemical characteristics, and additional confirmatory methods, as needed.

Molecular detection of *Mycoplasma genitalium* (*M. genitalium*), *Mycoplasma hominis* (*M. hominis*), *U. urealyticum*, *T. vaginalis*, *N. gonorrhoeae*, *C. trachomatis* and high-risk HPV was carried out on exfoliated cervical cells using a fully automated, CE-IVD-certified real-time PCR platform from Seegene Inc. (Seoul, South Korea), which integrates nucleic acid extraction, amplification and data analysis into a single streamlined workflow. The Allplex™ STI Essential Assay Q is a multiplex real-time PCR assay used in this study to detect key sexually transmitted pathogens, offering both qualitative identification and quantitative data for *M. hominis* and *U. urealyticum* to support appropriate diagnosis and management.

For HPV genotyping, the Allplex™ HPV HR Detection assay was employed. This multiplex PCR test simultaneously amplifies and identifies nucleic acids from 14 high-risk HPV types (16, 18, 31, 33, 35, 39, 45, 51, 52, 56, 58, 59, 66, and 68), along with an internal control, in a single reaction. The assay uses multiple fluorescent channels to differentiate individual genotypes and incorporates an internal control to monitor the entire process, from nucleic acid extraction to amplification. HPV genotypes were assigned based on fluorescence signal thresholds.

The study received ethical approval from the Ethics Committee of the Teaching Institute for Public Health of Split and Dalmatia County (Class: 500-01/15-01/6, No: 2181-103-01-15-1). All procedures were conducted in accordance with the principles outlined in the Declaration of Helsinki.

### Data Analysis

Data were analyzed using standard statistical approaches. The age variable was defined as a binary categorical variable, grouping women into two categories: ≤29 years and ≥30 years. This categorization was used consistently in the analysis as a nominal variable. The Kolmogorov–Smirnov test was used to assess the distribution of continuous variables. As the normality assumption was not met, non-parametric methods and categorical tests were applied accordingly. Descriptive statistics were calculated and presented for the overall study population.

Since some women were concurrently positive for multiple high-risk HPV genotypes, each genotype detection was treated as an individual line-level observation in the analysis. As a result, the dataset comprised a total of 1833 HPV entries, reflecting the cumulative number of genotype-specific detections rather than the number of individual participants. This approach allowed for a more detailed exploration of genotype-specific patterns and their associations with co-infecting microorganisms and age.

For the purposes of statistical analysis, high-risk HPV genotypes were categorized into four groups based on their established carcinogenic potential as outlined in the IARC Handbook and supported by data from large-scale epidemiological studies [21,22,23,24]. Specifically, HPV-16 was classified as the highest-risk genotype and analyzed as a distinct group due to its predominant role in cervical cancer. HPV-18 and HPV-45 were grouped together as the “18/45 group,” given their intermediate risk and joint classification in the literature. The remaining high-risk genotypes phylogenetically related to HPV-16 (types 31, 33, 35, 52, and 58) were grouped under “16-related” genotypes. Finally, genotypes with relatively lower carcinogenic risk (types 39, 51, 56, 59, 66, and 68) were categorized as the “lower-risk” group. This classification served as the basis for HPV group comparisons in our subsequent analyses.

To examine associations between categorical variables, including age group and specific pathogen detection, chi-square (χ^2^) tests of independence were conducted. Participants were classified into the aforementioned age groups (≤29 vs. ≥30 years) and appraised with either positive or negative test results for *C. trachomatis*, *M. hominis*, *M. genitalium*, *U. urealyticum*, *T. vaginalis*, *Candida albicans*, as well as a range of Gram-positive (*Staphylococcus aureus*, *Streptococcus agalactiae*) and Gram-negative (*Escherichia coli*, *Klebsiella pneumoniae*, *Morganella morganii*, *Pseudomonas aeruginosa*) pathogens. The same was carried out for high-risk HPV genotypes, which were grouped into four predefined categories: HPV 16 high-risk, HPV 18/45, HPV 16-related and lower-risk types. The strength of the association was evaluated based on the chi-square statistic and a corresponding *p*-value. Additionally, Spearman’s rank-order correlation was used to examine the association between age (as a continuous variable) and HPV genotype group, since the data did not meet the assumptions for parametric correlation analysis.

To explore whether the presence of selected microorganisms could predict HPV genotype group, a multinomial logistic regression analysis was performed. The dependent variable was the HPV type group, categorized as “HPV 16 high-risk”, “HPV 16-related”, and “lower-risk HPV”, with the “HPV 18/45” group serving as the reference category. The independent variables included the binary presence (positive vs. negative) of *C. trachomatis*, *M. genitalium*, *M. hominis*, *U. urealyticum* and *G. vaginalis*, as determined by microbiological and molecular diagnostics. Multinomial logistic regression was selected to model the log-odds of group membership relative to the reference group. Estimates, standard errors, Z-scores and *p*-values were obtained for each predictor across all outcome categories. Model fit was assessed using the deviance statistic, Akaike Information Criterion (AIC), and McFadden’s pseudo R^2^. Statistical significance was set at *p* < 0.05 (two-tailed).

All statistical analyses were performed using R (version 4.4) [25] and Jamovi software (version 2.6) [26]. The analysis incorporated several R packages, including car for applied regression modelling, ppcor for partial and semi-partial correlation analysis, and nnet for multinomial logistic regression. All packages were obtained from the CRAN repository.

## 3. Results

**Figure 1:**
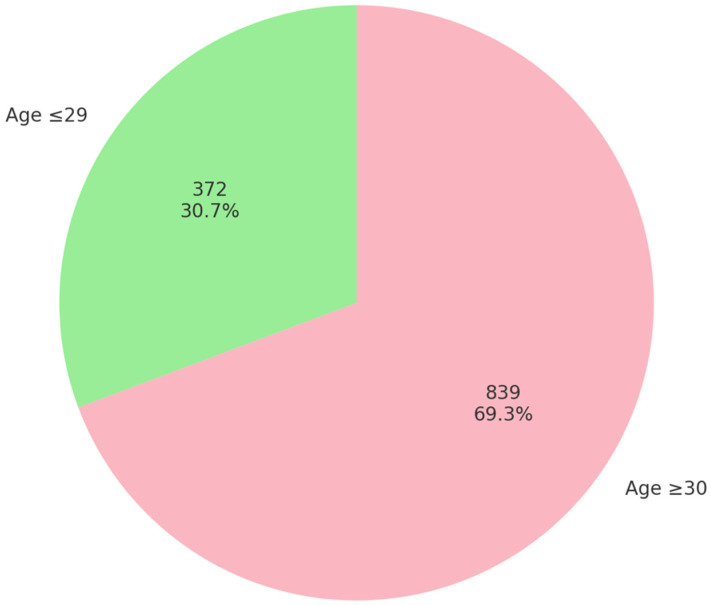
Age distribution of HPV-positive women included in the study (N = 1211).

In this study, in one-year period, a total of 3098 women were tested for high-risk HPV, with 1211 (39.09%) of them being positive. Among these, 39.9% were infected with multiple HPV types (483 out of 1211). When stratified by age, a positive result was found in 372 of 688 women aged ≤29 years (54.1%) and in 839 of 2410 women aged ≥30 years (34.8%). This difference was statistically significant (χ^2^ = 82.55, *p* < 0.001), indicating a markedly higher positivity rate in the younger age group. Among 1211 HPV-positive patients, there were a total of 1833 distinct HPV genotypes. The mean age of the study cohort (N = 1211) was 36.9 years, median was 35 years, mode 27 years, while the interquartile range (IQR) was 16.5 years. The age range was 63 years (the youngest age was 16 and the oldest age was 79 years). Out of the total number of HPV-positive women (N = 1211), 30.7% (372/1211) of them were ≤29, while 69.3% (839/1211) were ≥30 years old (Figure 1).

Out of the total number of HPV-positive women (N = 1211), the following genotype distribution was detected: HPV 16 in 282 (23.3%), HPV31 in 271 (22.4%), HPV 51 in 164 (13.5%), HPV 66 in 146 (12.1%), HPV 56 in 140 (11.6%), HPV 52 in 133 (11.0%), HPV 68 in 113 (9.3%) cases, HPV 18, HPV 58 in 98 cases (8.1%), HPV 59 in 97 (8.0%) cases, HPV 33 and HPV 39 each in 87 (7.2%) cases, HPV 45 in 72 (5.9%), and HPV 35 in 45 (3.7%) cases.

**Figure 2:**
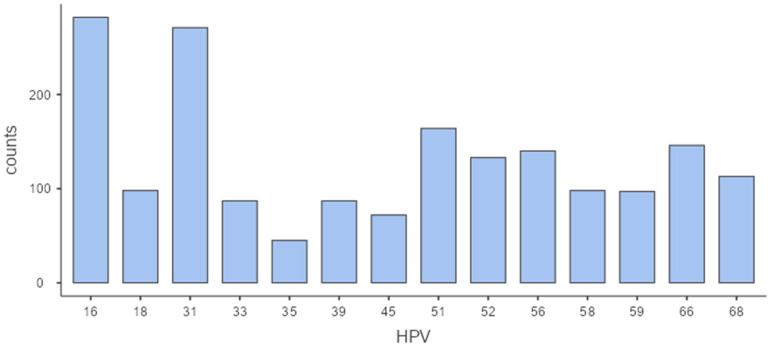
Bar graph displaying the frequency of each HPV genotype detected, with HPV 16 and HPV 31 being the most prevalent types (N = 1833).

Likewise, since a third of HPV-positive women were infected with multiple HPV genotypes, there were a total of 1833 distinct genotypes, among which the most frequently detected were HPV 16 (282 detections, 15.4%), HPV 31 (271 detections, 14.8%) and HPV 51 (164 detections, 8.9%). These were followed by HPV 66 (146; 8.0%), HPV 56 (140; 7.6%), HPV 52 (133; 7.3%) and HPV 68 (113; 6.2%). Less frequently detected types included HPV 18 (98; 5.3%), HPV 58 (98; 5.3%), HPV 59 (97; 5.3%), HPV 33 (87; 4.7%), HPV 39 (87; 4.7%), HPV 45 (72; 3.9%), as well as HPV 35 (45; 2.5%). Collectively, the top three genotypes (HPV 16, 31, and 51) accounted for nearly 40% of all HPV detections in our study. The distribution of all detected HPV genotypes in the study can be seen in Table 1 and Figure 2.

As already mentioned, among all HPV-positive women (N = 1211), 30.7% (372 individuals) were aged 29 or younger, while the remaining 69.3% (839 individuals) were aged 30 or older (χ^2^ = 82.55, *p* < 0.001). Statistical analysis revealed no statistically significant association between age group and HPV group (“HPV-16”, “18/45 group”, “16-related”, and “lower-risk” groups) positivity (χ^2^ = 0.859, *p* = 0.835), which suggests that the distribution of HPV genotype groups did not differ meaningfully between younger and older women in the sample (Table 2). Likewise, Spearman’s correlation analysis showed no significant association between age and HPV group positivity (Spearman’s rho = 0.014, *p* = 0.562), indicating that age was not correlated with the type of high-risk HPV infection.

Among the HPV-positive women (N = 1211), a total of 750 of them (234 ≤ 29 and 516 ≥ 30) were also tested for other pathogens in a multiplex panel, while 654 of women (198 ≤ 29 and 456 ≥ 30) were tested for a range of different aerobic/anaerobic cultivable bacteria and fungi—including *G. vaginalis*. Consequently, *C. trachomatis* was detected in 3.87% of cases (29/750), while *M. genitalium* was found in 1.47% (11/750) of them. *U. urealyticum* had a higher detection rate of 13.07% (98/750), as did *M. hominis*, detected in 11.46% (86/750) of women. *T. vaginalis* was detected in 5.33% of cases (4/750), while *N. gonorrheae* was not detected in our study cohort. *G. vaginalis* (a marker for cervico-vaginal dysbiosis) was observed in 17.28% (113/654) of the tested individuals. *C. albicans* was observed in 8.41% (55/654) of tested women, *S. agalactiae* in 3.06% (20/654), *E. coli* in 2.75% (18/654), *S. aureus* in 0.61% (4/654), *K. pneumoniae* in 0.31% (2/654), while *Morganella morganii* and *Pseudomonas aeruginosa* were detected in one woman each (0.15%).

There were statistically significant associations of some other pathogens with age groups; notably, *C. trachomatis* positivity was substantially higher among women aged 29 or younger (22/234; 9.61%) compared to those aged 30 or older (7/516; 1.40%) (χ^2^ = 28.07, *p* < 0.001), indicating that younger age was significantly associated with increased *C. trachomatis* detection in this cohort. Likewise, *S. agalactiae* was more frequently isolated in women aged 29 or younger, with 6.06% detection rate (12/198) compared to 1.75% (8/456) in women aged 30 or older (χ^2^ = 7.06, *p* = 0.029), suggesting a differing age-related distribution pattern in microbial findings. However, there were no statistically significant differences in relation to age groups for *U. urealyticum* (39/234 in 29 or younger; 59/516 in 30 or older) (χ^2^ = 3.56, *p* = 0.059), *M. hominis* (34/234 in 29 or younger; 52/516 in 30 or older) (χ ^2^= 2.92, *p* = 0.087), *M. genitalium* (4/234 in 29 or younger; 7/516 in 30 or older) (χ^2^ = 0.0029, *p* = 0.957) and *G. vaginalis* (30/198 in 29 or younger; 83/456 in 30 or older) (χ^2^ = 0.72, *p* = 0.397) positivity.

When comparing the HPV 16 high-risk group to the reference category (18/45), none of the predictors reached statistical significance, although *G. vaginalis* showed a borderline association (*p* = 0.073), suggesting a potential trend toward increased odds of membership in the HPV 16 group in women with dysbiosis. In contrast, statistically significant associations were found in the 16-related and lower-risk HPV groups. Specifically, *C. trachomatis* was negatively associated with being in the 16-related group (Estimate = −0.9583, *p* = 0.045), indicating that women with *C. trachomatis* had approximately 62% lower odds (OR ≈ 0.38) of belonging to this group compared to the 18/45 reference group. Similarly, *G. vaginalis* was positively associated with the 16-related group (Estimate = 0.9610, *p* = 0.029), with an odds ratio of approximately 2.6 (Table 3).

For the lower-risk HPV group, *C. trachomatis* was again a significant negative predictor (Estimate = −1.2722, *p* = 0.007), with an estimated 72% reduction in odds (OR ≈ 0.28) of being in the lower-risk group when *C. trachomatis* was present. *G. vaginalis* showed a near-significant positive association (Estimate = 0.8316, *p* = 0.055), suggesting a possible trend toward increased likelihood of lower-risk HPV infection in the presence of cervico-vaginal dysbiosis. Taken together, these findings suggest that *C. trachomatis* is more frequently detected in women infected with HPV 18/45 than in those with 16-related or lower-risk HPV genotypes. Conversely, *G. vaginalis* (as a marker of cervico-vaginal dysbiosis) appears more closely associated with non-18/45 HPV groups, particularly the 16-related group. Model fit statistics indicated limited explanatory power (Deviance = 2201, AIC = 2237, McFadden’s R^2^ = 0.00680) (Table 3).

## 4. Discussion

This retrospective study provides a detailed overview of the genotype-specific distribution of high-risk HPV in HPV-positive women from Southern Croatia, alongside its associations with selected sexually transmitted pathogens and microbial correlates. The most frequently detected genotypes were HPV 16, 31 and 51, collectively accounting for nearly 40% of all HPV genotype detections (and represented in almost 60% of all high-risk HPV-positive women). These findings are consistent with global and European trends, where HPV 16 remains the most prevalent and oncogenic type, which is also the case for Central and Eastern Europe [27,28]. Genotypes such as HPV 31 and 51 are also commonly reported in certain regional studies [29]. The relatively high prevalence of HPV 31 in our cohort further supports its classification as a high-risk genotype deserving focused clinical attention in screening and vaccination strategies.

The age is a known determinant of HPV acquisition and persistence [30,31], and we also showed how younger age is significantly linked with higher HPV prevalence. Several previous studies conducted in European, Chinese and North American populations also demonstrated that high-risk HPV prevalence consistently peaked in women under the age of 35 and declined with advancing age [29,32,33,34,35]. However, there is a lower body of literature on the interrelation between HPV types and different age categories. Our analysis did not reveal a statistically significant association between age groups and specific HPV genotype clusters, which aligns with some recent studies suggesting that the distribution of individual genotypes is not always age-dependent [36]. Such patterns may be influenced by the natural history of each genotype, with some (e.g., HPV 16) being more persistent and oncogenic, thereby remaining detectable over longer periods regardless of age [37,38]. Furthermore, this stability in genotype distribution across age groups could reflect regional transmission dynamics, vaccine coverage or cohort-specific risk behaviours that shape the local epidemiology of HPV—factors that were beyond the scope of this study.

When other pathogens were concerned, co-infections with *C. trachomatis* were significantly more frequent in women aged 29 younger in our analysis. This finding goes in line with the common assumption that younger women are more likely to harbour sexually transmitted infections (STIs) due to higher sexual activity and partner turnover [39], and many different studies show that the younger age group is disproportionately affected [40,41]. We also found a significant correlation of *S. agalactiae* positivity and age, with higher frequency in younger age groups. However, this association is not consistently reported in the literature, as some studies have observed higher colonization rates in older age groups [42]. In any case, our findings highlight the need for broader STI screening approaches that consider not only age, but also microbial co-factors that may act synergistically with HPV in promoting persistence and disease progression.

The present study confirms and expands upon earlier findings regarding the distribution of high-risk HPV genotypes in Southern Croatia. In the 2013 study by Kaliterna et al. [43], conducted on a cohort of 1160 women screened using Hybrid Capture 2 (HC2) with 406 (35%) HPV-positive cases, followed by PCR-based genotyping, HPV 16 emerged as the most prevalent genotype (in 30.8% cases), followed by HPV 18 (22.2%) and HPV 31 (6.7%) among the 406 high-risk HPV positive women. In contrast, our current study utilized a more sensitive multiplex real-time PCR assay and identified different HPV genotype distribution among 1211 HPV-positive women: HPV 16 as the most prevalent genotype as well (in 23.3% of cases), followed by HPV 31 (22.4%) and HPV 51 (13.5%)—showing higher prevalence of certain genotypes compared to the 2013 data. This marked increase in HPV 31 may be attributed to the methodological improvements in genotyping sensitivity and resolution, as well as possible changes in local epidemiological dynamics over the past decade. Additionally, HPV 18, which was the second most common genotype in 2013, was less dominant ten years later in this study.

Present study focused exclusively on high-risk HPV-positive women, enabling more detailed genotype-specific analysis and assessment of co-infection patterns. Likewise, a study conducted in Northern Croatia (Zagreb County) on cervical samples found HPV 16 being the most commonly detected high-risk genotype, present in 33.3% of HPV-positive samples, followed by HPV 31 in 14.0% of HPV-positive samples [44]. This comparison highlights the importance of study design and target population in shaping observed genotype prevalence patterns. In any case, the findings suggest a broader and more diverse HPV genotype landscape in different regions of Croatia today, underscoring the need for ongoing epidemiological surveillance with high-resolution molecular diagnostics approaches.

The findings can also be contextualized by comparing them with a recent study conducted in the same geographic region in 2023 [20], which investigated HPV prevalence and its association with genital microbiota in a general population of asymptomatic women. The mentioned study and our study both aimed to appraise the role of microbial co-infections (most notably with *C. trachomatis* and *G. vaginalis*); however, our study further disentangles these associations by employing multinomial regression to demonstrate genotype-specific relationships. For instance, *C. trachomatis* showed a significant negative association with 16-related and lower-risk HPV groups, whereas *G. vaginalis* was positively associated with 16-related types. Unlike the 2023 study, which focused primarily on overall HPV positivity, this work aimed to provide insights into how distinct microbial patterns may influence the distribution of specific oncogenic genotypes, contributing, in turn, to a more nuanced understanding of HPV–microbiome interactions in cervical cancer risk stratification.

While HPV 16 remains the most commonly detected high-risk genotype, our findings align with broader global and European trends that highlight the growing relevance of other high-risk HPV types. For example, in the general European population, HPV 31 and HPV 18 are frequently reported following HPV 16 [38]. In our Southern Croatian cohort, after HPV 16 (detected in 23.3% HPV-positive women), HPV 31 and HPV 51 were particularly prevalent (in 22.4% and 13.5% cases, respectively), along with notable detection rates for HPV 66 (12.1%), HPV 56 (11.6%) and HPV 52 (11.0%). Some parallels can also be drawn with a recent Greek study, which identified HPV 31, HPV 66, HPV 56 and HPV 51 as the most common high-risk HPV types after HPV 16 in their study population [39]. In the northern region of Portugal, the most common high-risk HPV genotypes among high-risk HPV positive women were HPV 16 (17.5%), followed closely by HPV 39 (16.7%), HPV 31 (15.0%), HPV 68 (13.2%), HPV 52 (10.7%) and HPV 51 (10.6%) [45]. These aforementioned genotypes—particularly HPV 51, HPV 56 and HPV 66—are not covered by the currently available nine-valent HPV vaccine [46], emphasizing the need for ongoing regional surveillance. Our data also underscore the importance of monitoring shifts in genotype distribution to inform local vaccination strategies and to support the potential development of next-generation multivalent vaccines tailored to regional epidemiological profiles.

A recent systematic review analyzing 55 European studies on high-grade cervical intraepithelial neoplasia (CIN 2+) found that HPV 16 was consistently the most prevalent high-risk genotype both before and after treatment, followed by HPV 18, HPV 31 and HPV 58 [47]. In one study on 216 women that was included in the aforementioned systematic review, HPV 16 was detected in 55.5% of cases pre-treatment and remained in 7.9% six months after conization, while HPV 31 and HPV 52 also showed notable persistence [47]. The systematic review revealed that although overall HPV positivity declined substantially after excisional therapy, certain genotypes such as HPV 16 and HPV 58 demonstrated a greater tendency to persist, contributing to the risk of recurrent disease [47]. The findings emphasize the need for genotype-specific post-treatment monitoring, considering the fact that the persistence of particular high-risk types may influence long-term outcomes and inform future vaccination and screening strategies.

This study has several limitations that should be acknowledged. The retrospective and cross-sectional design limits the ability to infer causality between specific microbial co-infections and HPV genotype distribution. While the study included a relatively large sample of HPV-positive women, it was conducted in a single geographic region, which may limit the generalizability of findings to other populations in Croatia or neighbouring countries. Then, the analysis relied on cervico-vaginal swabs collected during routine gynecological examinations, but lacked accompanying clinical data such as cytological results or specific behavioural risk factors (e.g., number of sexual partners, contraceptive use), which could further contextualize the associations observed. And although molecular testing platforms used were CE-IVD-certified and capable of detecting multiple pathogens, the microbiological component did not assess the broader composition of the cervico-vaginal microbiota, limiting insights into complex microbial community interactions beyond selected pathogens. Furthermore, the study did not include data on participants’ comorbidities, fertility status, prior HPV infection history, HPV vaccination status and/or use of concomitant medications, all of which could influence HPV genotype prevalence, co-infection rates and immune response. These gaps may introduce potential bias in data interpretation and limit the ability to draw definitive conclusions about observed associations. Finally, the grouping of HPV genotypes into four predefined categories may obscure more subtle differences among individual types, and the relatively low pseudo R^2^ value of the regression model indicates limited explanatory power, suggesting that other unmeasured variables may contribute to the observed genotype distributions.

## 5. Conclusions

Our study offered a detailed snapshot of the genotype-specific distribution of high-risk HPV and its microbial correlates in HPV-positive women from Southern Croatia, more specifically from Split and Dalmatia County. HPV 16, 31, and 51 emerged as the most prevalent high-risk genotypes, reinforcing global and regional trends and emphasizing the need for ongoing surveillance beyond HPV 16 and 18. Importantly, we identified significant associations between cervico-vaginal microorganisms and HPV genotype groupings, which underscore the value of integrating extended HPV genotyping with microbial screening in routine gynecological care to yield clinically relevant insights into co-infection patterns and understanding potential implications for cervical cancer risk.

Despite limitations related to its retrospective and single-region design, we believe the study contributes to a more nuanced understanding of HPV epidemiology and highlights the importance of context-specific data. From a policy perspective, the results support the expansion of national cervical cancer screening programmes to include extended genotyping for high-risk HPV types beyond those covered by current vaccines. The high prevalence of non-vaccine genotypes underscores the need to monitor shifts in genotype distribution that may impact long-term vaccine effectiveness. This also has important implications for clinical oncology practice, as understanding the patterns of HPV co-infection may inform risk stratification, improve the interpretation of screening results, and also guide the development of more targeted prevention and management strategies for HPV-associated cervical lesions.

## Figures and Tables

**Table 1:** Distribution of detected HPV genotypes in the study cohort.

HPV Genotype	Counts	% of Total High-Risk HPV-Positive Women (N = 1211)	% of Total Genotypes (N = 1833)
16	282	23.3%	15.4%
18	98	8.1%	5.3%
31	271	22.4%	14.8%
33	87	7.2%	4.7%
35	45	3.7%	2.5%
39	87	7.2%	4.7%
45	72	5.9%	3.9%
51	164	13.5%	8.9%
52	133	11.0%	7.3%
56	140	11.6%	7.6%
58	98	8.1%	5.3%
59	97	8.0%	5.3%
66	146	12.1%	8.0%
68	113	9.3%	6.2%

**Table 2:** Distribution of high-risk HPV genotype groups across age categories.

	High-Risk HPV Group				
Age Group	18/45	16 High Risk	16-Related	Lower Risk	Total
29 or under	109	183	393	467	1152
30 or over	61	99	241	280	681
Total	170	282	634	747	1833
*p*-value					0.835

**Table 3:** Multinomial logistic regression predicting HPV genotype group membership based on the presence of other pathogens.

HPV Groups	Predictor	Estimate	SE	Z	*p*-Value	OR
16 high-risk—18/45	Intercept	0.5626	0.169	3.336	<0.001	1.755
	CT:					
	P—N	−0.6578	0.531	−1.239	0.215	0.518
	MG:					
	P—N	−0.3040	0.946	−0.321	0.748	0.738
	Mh:					
	P—N	−0.1740	0.471	−0.369	0.712	0.840
	Uu:					
	P—N	−0.0786	0.407	−0.193	0.847	0.924
	GV:					
	P—N	0.8512	0.475	1.791	0.073	2.343
16-related—18/45	Intercept	1.3876	0.151	9.195	<0.001	4.005
	CT:					
	P—N	−0.9583	0.479	−2.000	0.045	0.384
	MG:					
	P—N	−1.0822	0.941	−1.150	0.250	0.339
	Mh:					
	P—N	−0.1778	0.425	−0.418	0.676	0.837
	Uu:					
	P—N	−0.2572	0.371	−0.694	0.488	0.773
	GV:					
	P—N	0.9619	0.441	2.180	0.029	2.617
Lower risk—18/45	Intercept	1.6403	0.147	11.134	<0.001	5.157
	CT:					
	P—N	−1.2722	0.474	−2.684	0.007	0.280
	MG:					
	P—N	−0.4983	0.822	−0.606	0.544	0.608
	Mh:					
	P—N	0.1108	0.408	0.271	0.786	1.117
	Uu:					
	P—N	−0.1185	0.357	−0.332	0.740	0.888
	GV:					
	P—N	0.8316	0.434	1.916	0.055	2.297

The table displays the results of a multinomial logistic regression analysis assessing the association between selected microorganisms and HPV genotype group membership. The HPV 18/45 group served as the reference category. For each comparison (16 high-risk, 16-related and lower-risk HPV groups vs. 18/45), regression coefficients (estimates), standard errors (SE), Z-values, *p*-values and odds ratios (OR) are presented. “P—N” denotes “Positive—Negative”; “CT” denotes *“Chlamydia trachomatis*”; “MG” denotes “*Mycoplasma genitalium*”; “MH” denotes “*Mycoplasma hominis*”; “Uu” denotes “*Ureaplasma urealyticum*”; “GV” denotes “*Gardnerella vaginalis*”.

## Data Availability

Original dataset available upon reasonable request from the authors.

## Abbreviations

The following abbreviations are used in this manuscript:

AIC	Akaike Information Criterion
CE-IVD	Conformité Européenne—In Vitro Diagnostic
CIN 2	Cervical Intraepithelial Neoplasia Grade 2
CIN 3	Cervical Intraepithelial Neoplasia Grade 3
CT	*Chlamydia trachomatis*
DALY	Disability-Adjusted Life Year
GV	*Gardnerella vaginalis*
HPV	Human Papillomavirus
HR	High Risk
IQR	Interquartile Range
MG	*Mycoplasma genitalium*
MH	*Mycoplasma hominis*
NG	*Neisseria gonorrhoeae*
OR	Odds Ratio
Pap	Papanicolaou
PCR	Polymerase Chain Reaction
SE	Standard Error
STI	Sexually Transmitted Infection
UU	*Ureaplasma urealyticum*
TV	*Trichomonas vaginalis*
